# Atypical bleeding following blepharoplasty associated with curcumin supplementation: a case report

**DOI:** 10.1093/jscr/rjag1019

**Published:** 2026-11-04

**Authors:** Mayte Rodrigues da Cunha, Marco Tulio Rodrigues da Cunha

**Affiliations:** Department of Basic Sciences, Saint James School of Medicine, Cane Hall Road, Arnos Vale, Saint George Parish, VC0280, Saint Vincent and the Grenadines; Department of Plastic Surgery, Universidade Federal do Triângulo Mineiro (UFTM), Avenida Frei Paulino, 30, Bairro Abadia, Uberaba, Minas Gerais, 38025-180, Brazil

**Keywords:** blepharoplasty, postoperative hematoma, curcumin, platelet function, perioperative bleeding, dietary supplements

## Abstract

Blepharoplasty is generally associated with a low complication rate; however, postoperative hematoma may result in significant morbidity. We report a 39-year-old male who developed atypical bilateral bleeding following lower eyelid blepharoplasty despite normal preoperative prothrombin time and partial thromboplastin time. The patient reported ongoing curcumin supplementation that had not been discontinued before surgery. Within hours of the procedure, progressive bilateral swelling developed, and clinically significant hematomas were present on postoperative Day 1. Prompt drainage with placement of a Penrose drain was performed, followed by observation and intravenous tranexamic acid. The postoperative course was further complicated by substantial bleeding after minor periocular trauma requiring re-suturing. Given the normal routine coagulation studies and reported antiplatelet effects of curcumin, altered platelet function was considered a potential contributor. This case highlights the importance of including dietary supplements in preoperative medication assessment.

## Introduction

Blepharoplasty is one of the most commonly performed cosmetic surgical procedures and is generally associated with a low rate of complications. Postoperative hematoma, although uncommon, remains one of the most clinically significant adverse events due to the potential for orbital compression and visual compromise if not promptly recognized and managed [1, 2].

Perioperative bleeding risk assessment traditionally focuses on anticoagulant and antiplatelet medications. However, over-the-counter supplements may also influence hemostasis and may be overlooked during preoperative evaluation [3]. Curcumin, the principal bioactive component of *Curcuma longa* (turmeric), is widely used as a dietary supplement and has been reported to exert antiplatelet effects, including modulation of platelet activation and aggregation [4–8]. These effects may be particularly relevant in the perioperative setting, as alterations in platelet function may not be reflected by routine coagulation studies such as prothrombin time (PT) and partial thromboplastin time (PTT) [9].

We present a case of atypical postoperative bleeding following lower eyelid blepharoplasty in a patient with ongoing curcumin supplementation despite normal preoperative PT and PTT.

## Case report

A 39-year-old male presented for lower eyelid blepharoplasty (Fig. 1). His medical history included severe COVID-19 requiring hospitalization and anticoagulation with rivaroxaban, discontinued more than 1 year before surgery. Medications included quetiapine, lamotrigine, lithium carbonate, venlafaxine, and lisdexamfetamine. He also reported regular use of supplements including a multivitamin complex, magnesium, curcumin, coenzyme Q10, creatine, and glutamine, as well as exogenous testosterone. Curcumin had not been discontinued before surgery. No personal history of bleeding disorders was reported.

**Figure 1:**
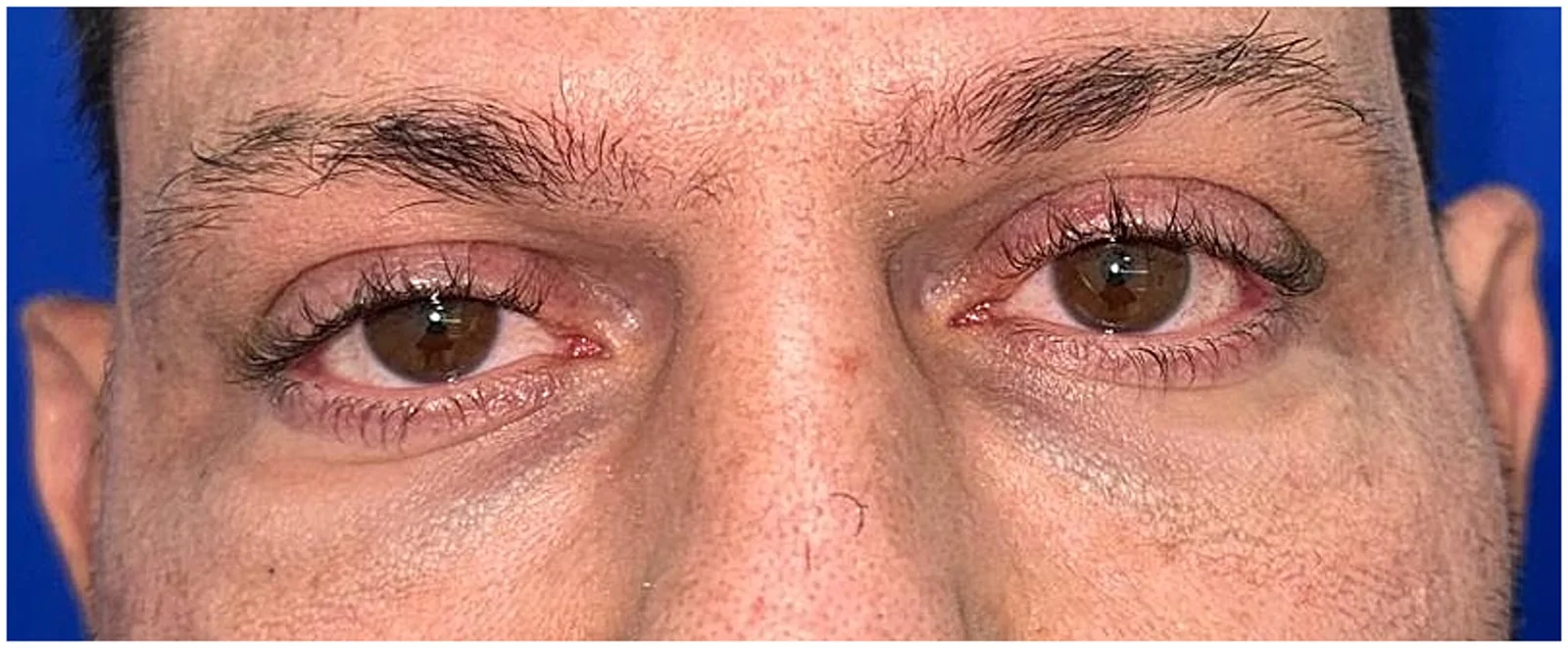
Preoperative appearance prior to lower eyelid blepharoplasty.

Preoperative evaluation on 24 September 2025, including PT and PTT, was within normal limits, and the patient was cleared by cardiology and anesthesia. Lower eyelid blepharoplasty was performed on 26 September 2025, without intraoperative complications.

Within hours, after intraoperative vasoconstriction subsided, atypical bleeding and progressive bilateral swelling developed. On postoperative Day 1, clinically significant bilateral periorbital hematomas were present, more pronounced on the right (Fig. 2). Prompt drainage and wound cleaning with placement of a Penrose drain were performed under local anesthesia (Fig. 3). The patient was admitted overnight for observation and received intravenous tranexamic acid.

**Figure 2:**
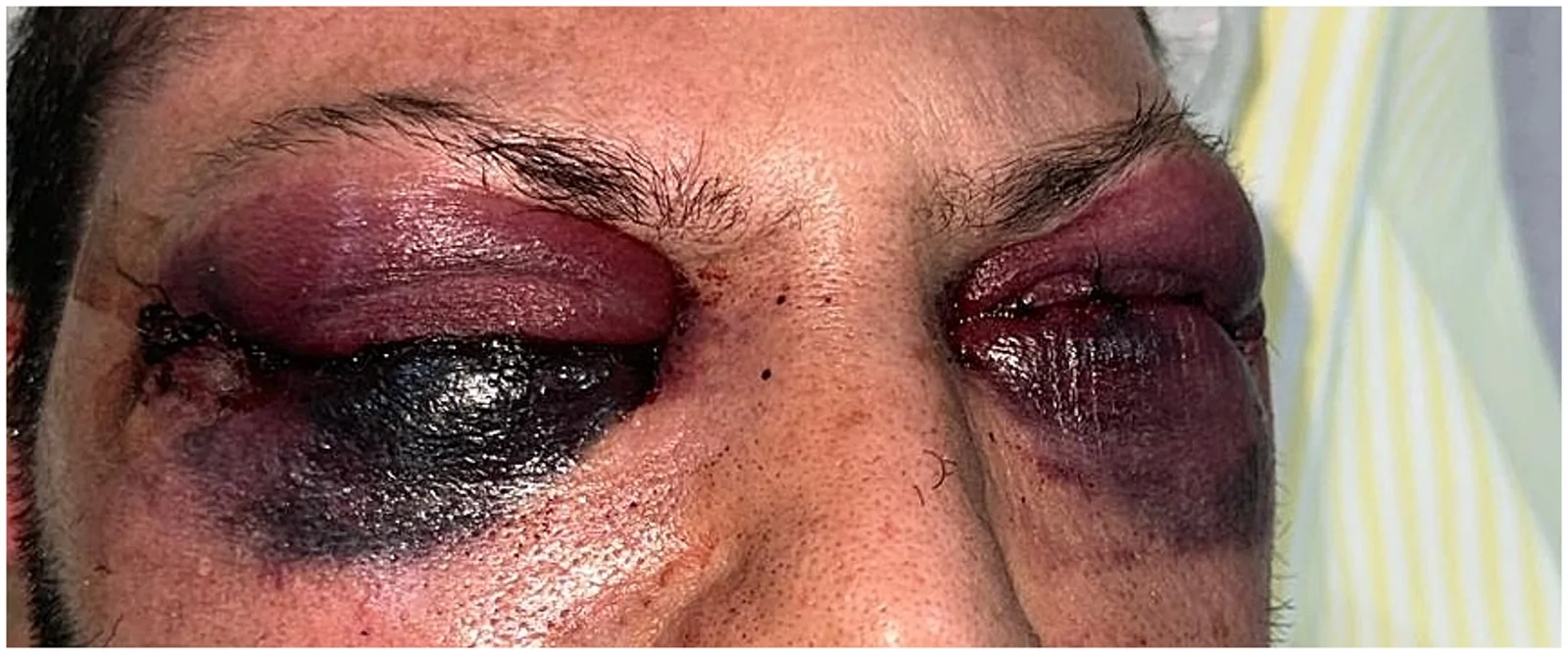
Postoperative Day 1: bilateral hematomas.

**Figure 3:**
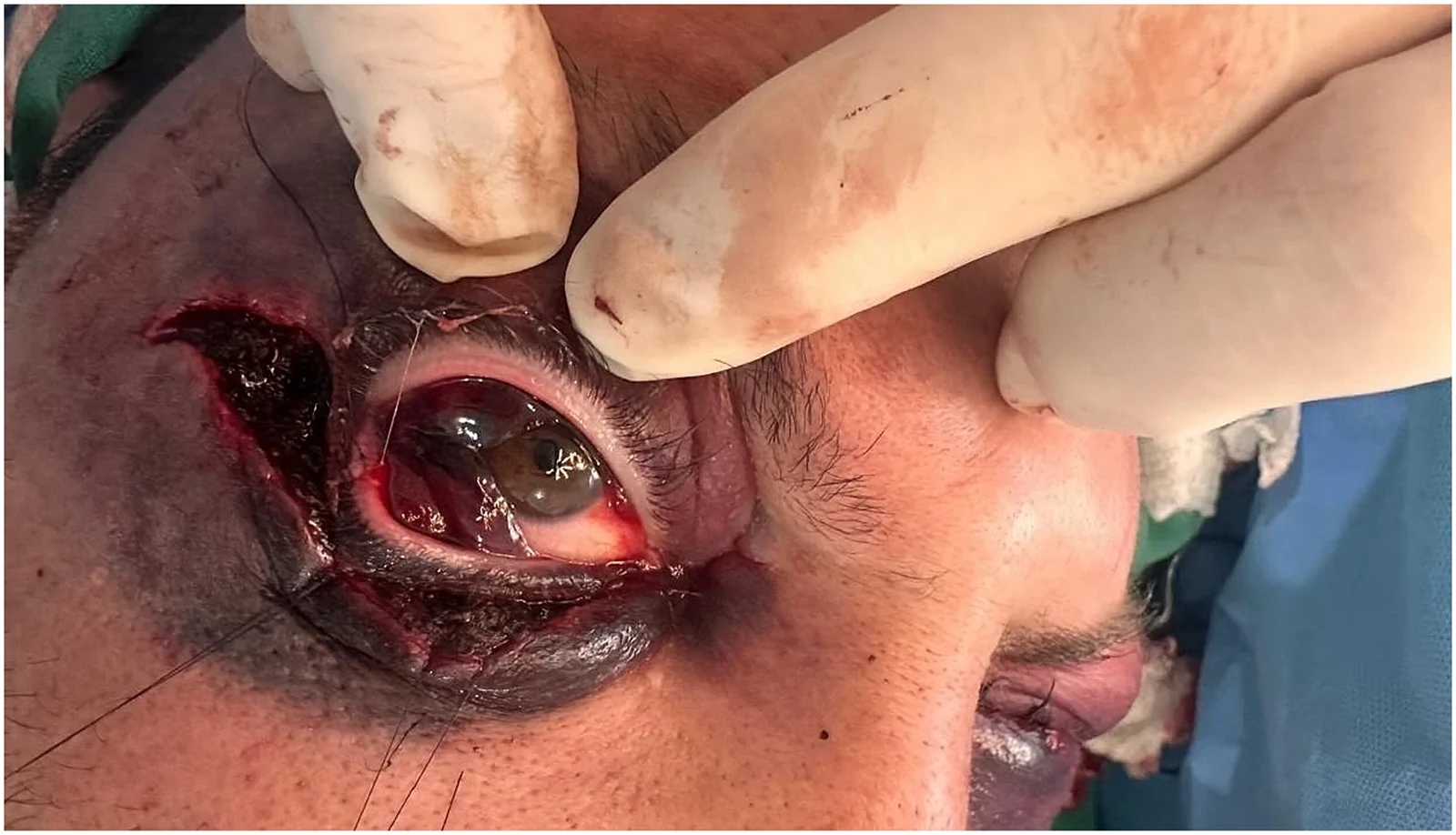
Intraoperative appearance of the right periocular region during drainage of the postoperative hematoma.

The drain was removed on 29 September, with subsequent improvement in edema and ecchymosis. On 8 October, the patient sustained minor left periocular trauma after accidentally striking his face during the night, resulting in wound dehiscence and substantial bleeding that appeared disproportionate to the trauma. Re-suturing was performed under local anesthesia. Subsequent follow-up demonstrated favorable healing. At final follow-up on 6 January 2026, the patient reported no pain or epiphora and was satisfied with the surgical outcome (Fig. 4).

**Figure 4:**
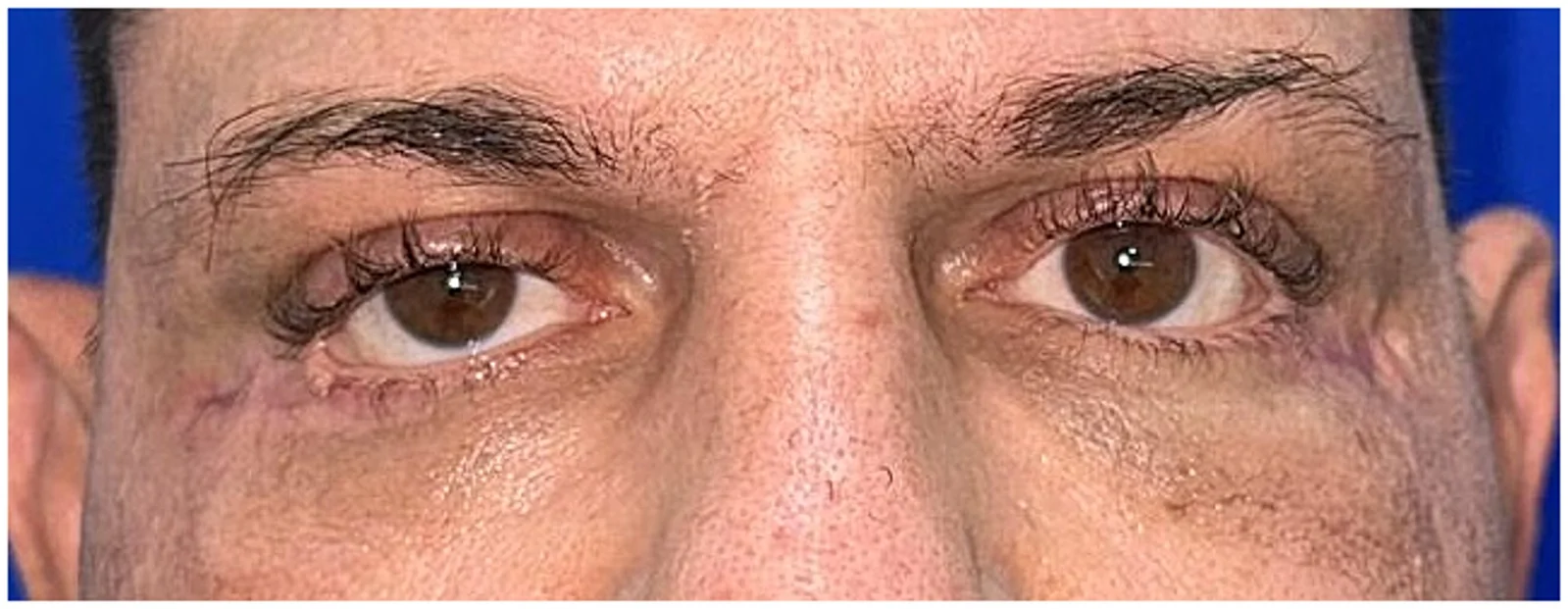
Final follow-up demonstrating resolution of edema with mild residual asymmetry.

## Discussion

Postoperative hematoma following blepharoplasty requires prompt recognition because of the potential for orbital compression and visual compromise [1, 2]. This case raises the possibility that perioperative dietary supplement use may represent an overlooked contributor to bleeding risk.

Curcumin has demonstrated effects on platelet activity, including inhibition of platelet activation and aggregation [4–8]. In this patient, supplementation continued through surgery despite normal PT and PTT. Because these tests primarily assess components of the coagulation cascade and do not directly evaluate platelet aggregation, normal results do not exclude abnormalities of platelet function [9]. The combination of normal routine coagulation studies, ongoing curcumin exposure, and substantial postoperative bleeding therefore raises the possibility of a platelet-mediated contribution, although platelet function testing was not performed.

The subsequent substantial bleeding following relatively minor periocular trauma represents a second clinically notable bleeding episode. However, other potential contributors must be considered. The patient was also taking venlafaxine and a multivitamin complex containing vitamin E, both of which may influence hemostasis [10, 11], and had a remote history of rivaroxaban therapy. These concurrent exposures prevent attribution of the bleeding specifically to curcumin.

This report is limited by its single-patient design, absence of platelet function testing, and unavailable information regarding the exact dose and formulation of the supplement. Nevertheless, the continued perioperative use of curcumin and its reported effects on platelet activity make it a clinically relevant consideration. This case highlights the importance of obtaining a comprehensive preoperative history that includes dietary and herbal supplements, particularly those with potential effects on hemostasis.
